# Selective synthesis and crystal structures of manganese(I) complexes with a bi- or tridentate terpyridine ligand

**DOI:** 10.1107/S2056989020008178

**Published:** 2020-06-26

**Authors:** Kosei Wadayama, Tsugiko Takase, Dai Oyama

**Affiliations:** a Graduate School of Science and Engineering, Fukushima University, 1 Kanayagawa, Fukushima 960-1296, Japan; b Department of Natural Sciences and Informatics, Fukushima University, 1, Kanayagawa, Fukushima 960-1296, Japan

**Keywords:** crystal structure, manganese(I) complex, terpyridyl ligand, distinct coordination mode, disorder

## Abstract

The crystal structures of two manganese(I) complexes with a different coordination mode of the supporting ligand are reported: *fac*-bromido­tricarbon­yl(4′-phenyl-2,2′:6′,2′′-terpyridine-κ^2^ *N*,*N*′)manganese(I), [MnBr(C_21_H_15_N_3_)(CO)_3_], **I**, and *cis*-bromido­dicarbon­yl(4′-phenyl-2,2′:6′,2′′-terpyridine-κ^3^ *N*,*N*′,*N*′′)manganese(I), [MnBr(C_21_H_15_N_3_)(CO)_2_], **II**. In both complexes, the manganese(I) atom is coordinated by terminal carbonyl ligands, a bromide ion, and a 4′-phenyl-2,2′:6′,2′′-terpyridine ligand within a distorted octa­hedral environment. In **I**, the metal ion is facially coordinated by three carbonyl ligands and the terpyridine ligand binds in a bidentate fashion. The non-coordinating nitro­gen atom in the terpyridine ligand is positioned on the side opposite to the bromido ligand. In **II**, the metal ion is coordinated by two carbonyl ligands in a *cis* configuration and the terpyridine ligand binds in a tridentate fashion; notably, one carbonyl and the *trans* bromido ligand are mutually disordered over two positions. In **I**, the complex mol­ecules are linked by C—H⋯Br hydrogen bonds. In **II**, aromatic π–π contacts are present, as well as pairs of C—H⋯Br and C—H⋯O hydrogen bonds.

## Abstract 2

The structural comparison of two Mn carbonyl complexes comprising a terpyridine derivative engaged in bidentate or tridentate coordination of the central Mn^I^ atom is reported.

## Chemical context   

Carbonyl­manganese(I) complexes with polypyridyl ligands are of particular inter­est as novel active mol­ecules that are able to release CO in response to photoirradiation (Carrington *et al.*, 2013 ▸; Chakraborty *et al.*, 2014 ▸; Jimenez *et al.*, 2015 ▸) or as electrocatalysts of CO_2_ reduction (Grills *et al.*, 2018 ▸; Stanbury *et al.*, 2017 ▸). Among these compounds, studies have concentrated mainly on tricarbonyl complexes comprising bidentate polypyridyl supporting ligands; by contrast, only few reports exist on dicarbonyl complexes bearing tridentate ligands (Compain *et al.*, 2015 ▸; Machan & Kubiak, 2016 ▸). In fact, even though the typically tridentate ligands 2,2′:6′,2′′-terpyridine and derivatives thereof coordin­ate to an Mn^I^ ion, the majority of them bind the metal ion in a bidentate manner (Compain *et al.*, 2014 ▸; Moya *et al.*, 2001 ▸).

As indicated by the results of studies focusing on the comparison between carbonyl­manganese complexes containing bidentate and tridentate terpyridines (Compain *et al.*, 2015 ▸; Machan & Kubiak, 2016 ▸), investigating the relationship between reactivity and mol­ecular structure is a key research objective. However, comparing these two systems experimentally is difficult, particularly considering that available structural data on complexes comprising tridentate terpyridine ligands are quite scarce.
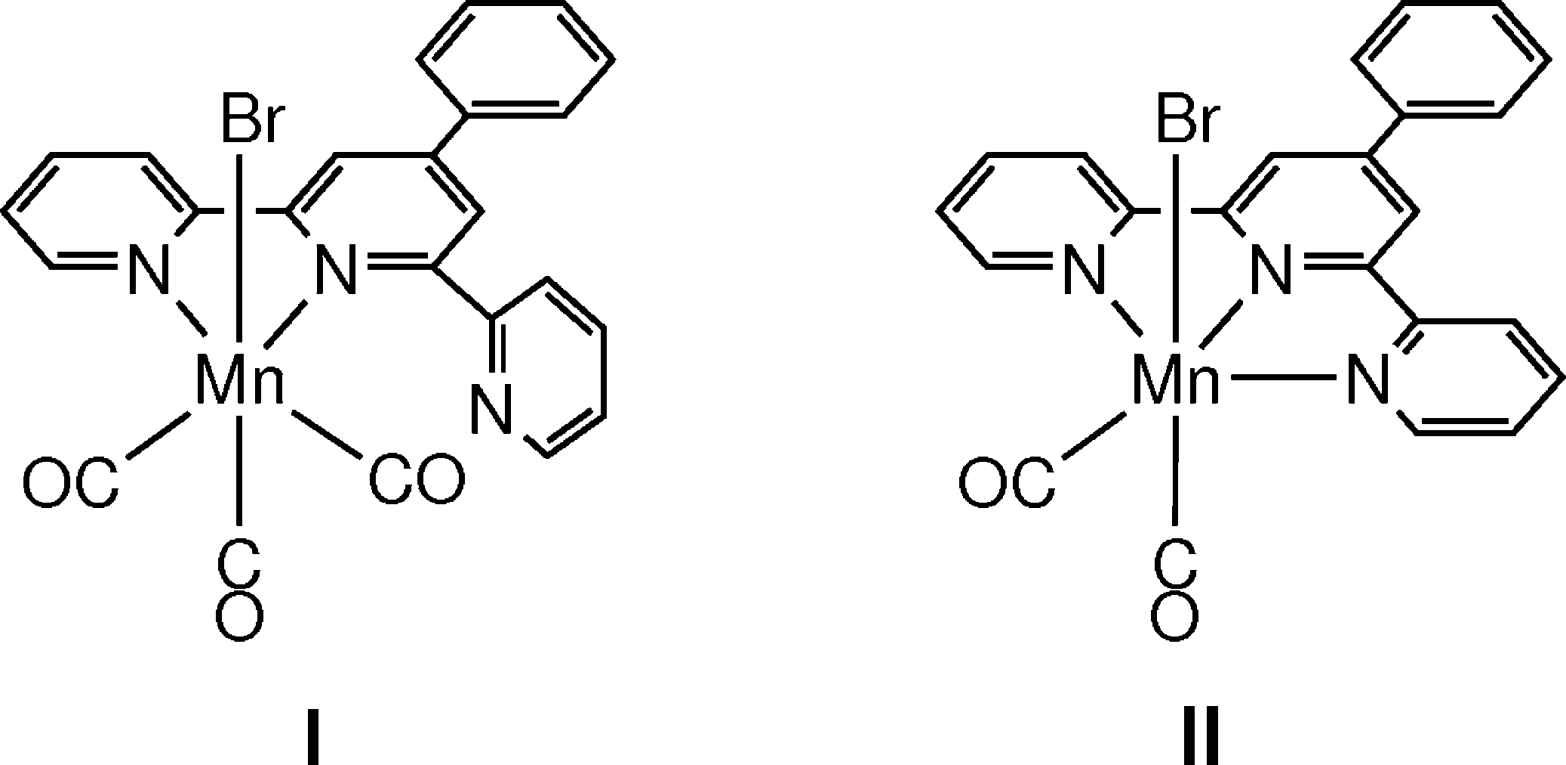


Herein, we report the structural characterization of complex *fac*(CO)-[Mn(tpyPh-κ^2^ *N*,*N*′)(CO)_3_Br] (**I**; tpyPh = 4′-phenyl-2,2′:6′,2′′-terpyridine) comprising a bidentate terpyridine-based ligand, which has been synthesized by Moya *et al.* (2001 ▸), and the synthesis and characterization of the corresponding complex *cis*(CO)-[Mn(tpyPh-κ^3^ *N*,*N*′,*N*′′)(CO)_2_Br] (**II**), whereby the same terpyridine-based ligand is tridentate.

## Structural commentary   

**Figure 1:**
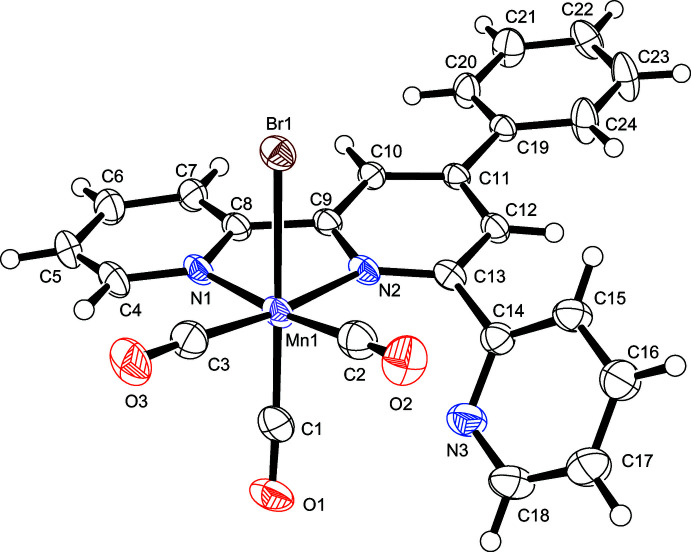
The mol­ecular structure of compound **I**, with atom labeling and displacement ellipsoids drawn at the 50% probability level.

The mol­ecular structures of compounds **I** and **II** are displayed in Figs. 1 ▸ and 2 ▸, respectively. Although **I** was prepared by Moya *et al.* (2001 ▸), its structure has not previously been determined. In **I** and **II**, the manganese(I) atoms exhibit distorted octa­hedral coordination environments, similar to those reported for other structurally related complexes (Compain *et al.*, 2014 ▸, 2015 ▸). In **I**, the *fac* configuration of the three CO ligands around the central manganese(I) atom is in agreement with the IR data of the complex and similar to those previously reported for complexes of this type (Compain *et al.*, 2014 ▸, 2015 ▸). As can be evinced from Fig. 1 ▸, the terpyridine ligand exhibits a bidentate coordination with respect to the central Mn^I^ atom, so that one of the outer pyridyl rings remains outside the coordination sphere. The corresponding non-coordinating N atom, N3, is positioned on the side opposite to the Br atom. As a result, the torsion angle between the coordinating and non-coordinating pyridyl rings in **I** (N2—C13—C14—N3) is much smaller [47.9 (3)°] than those reported for related Mn^I^ complexes with bidentate terpyridine derivatives (Compain *et al.*, 2014 ▸, 2015 ▸). The non-coordinating N atom is positioned in proximity of the equatorial carbonyl ligand (C2≡O2), with a short value for the inter­atomic distance between C2 and N3 [2.900 (4) Å]. Since this distance is considerably shorter than the sum of the two atoms’ van der Waals radii (3.25 Å; Bondi, 1964 ▸), evidence suggests that an inter­action exists between the free pyridine and the adjacent CO ligand. This inter­action may explain the observation that the Mn1—C2 distance [1.840 (3) Å] is longer than the other two corresponding distances in **I** [Mn1—C1 = 1.805 (3) and Mn1—C3 = 1.796 (3) Å].

The crystal structures of Mn^I^ dicarbonyl complexes with tridentate terpyridines have very rarely been reported (Compain *et al.*, 2015 ▸), because of the instability in solution of compounds of this type. In **II**, the carbonyl ligands are in *cis* configuration, again in accordance with IR data. Differently from **I**, in **II** the Mn^I^ ion is coordinated by a tridentate terpyridyl ligand, as well as two CO ligands and a Br^−^ ion. Only the central Mn—N2 bond is slightly shortened (by ∼0.05 Å) as a result of geometric constraints. In contrast to **I**, where no disorder is observed, in **II** one of the CO ligands (C2≡O2) and the Br^−^ ligand are mutually disordered over two positions. The dihedral angle between the phenyl pendant and the central pyridyl ring in **II** is slightly larger than the corresponding angle in **I**. Specifically, the C10—C11—C19—C20 torsion angle has a value of −19.3 (5)° in **II** and −9.9 (4)° in **I**, but both values indicate an essential quasi-coplanarity. Notably, the extended conjugation made possible by the mentioned quasi-planarity may contribute to an increased stability of these compounds.

## Supra­molecular features   

**Figure 3:**
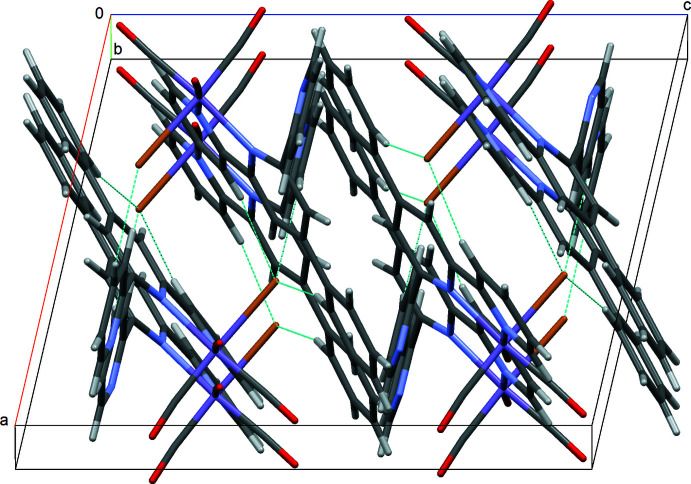
The crystal packing of compound **I** with C—H⋯Br hydrogen bonds shown as dashed lines.

In the crystal structure of **I**, complex mol­ecules display three kinds of C—H⋯Br hydrogen bonds (*i.e*., between the Br^−^ ligand and the C—H groups in the coordinating pyridyl ring, the free pyridyl ring, and the phenyl pendant), forming a three-dimensional supra­molecular structure (Table 1 ▸ and Fig. 3 ▸).

**Figure 4:**
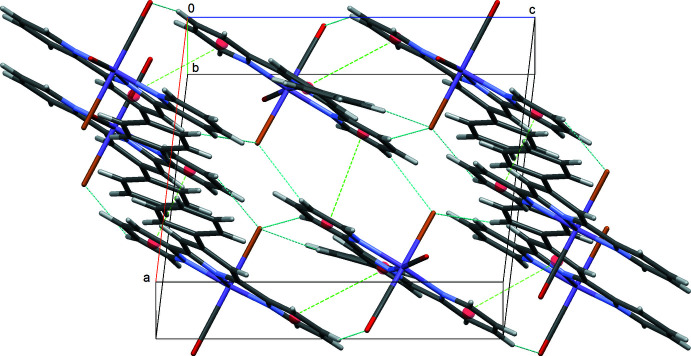
The crystal packing of compound **II** with C—H⋯Br and C—H⋯O hydrogen bonds (blue) and π–π contacts (green) shown as dashed lines; ring centroids are shown as red spheres.

In the crystal structure of **II**, weak C—H⋯Br and C—H⋯O hydrogen bonding inter­actions (Table 2 ▸) exist between the terpyridyl ligand and the disordered CO/Br ligands. Additional π–π inter­actions [*Cg*3⋯*Cg*2^iv^ = 4.000 (2) and *Cg*1⋯ *Cg*1^i^ = 4.128 (3) Å; *Cg*1, *Cg*2 and *Cg*3 are the centroids of the N1/C4–C8, N2/C9–C13 and N3/C14–C18 rings, respectively; symmetry codes: (i) 1 − *x*, −*y*, 2 − *z*; (iv) *x*, −*y* + 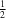, *z* − 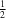] consolidate the crystal packing. These inter­actions lead to the formation of a three-dimensional network structure (Fig. 4 ▸).

## Database survey   

With respect to manganese(I) complexes with a tridentate terpyridine derivative ligand of the form *cis*(CO)-[Mn(tpy*R*)(CO)_2_Br], only a single structure, whereby *R* = *p*-tolyl, has been reported (Compain *et al.*, 2015 ▸). In contrast, some structures of bidentate terpyridine derivative-coordinated manganese(I) complexes have been reported by Compain *et al.* (2014 ▸, 2015 ▸).

## Synthesis and crystallization   

All the manganese(I) complexes were handled and stored in the dark to minimize exposure to light. Compound **I** was synthesized as described by Moya *et al.* (2001 ▸). The compound thus obtained proved to be analytically and spectroscopically pure (as determined by microanalysis, IR, UV–vis, and ^1^H NMR data). Crystals suitable for use in X-ray diffraction experiments were grown by vapor diffusion of diethyl ether into an acetone solution of **I**.

For the synthesis of compound **II**, bromido­penta­carbonyl­manganese(I) (30 mg, 0.11 mmol) and 4′-phenyl-2,2′:6′,2′′-terpyridine (31 mg, 0.10 mmol) were dissolved in an acetone–water mixture (20/30 ml). The solution thus obtained was refluxed for 24 h; the solvent was then evaporated under reduced pressure, and the resulting solid was placed in diethyl ether (50 ml); the resulting mixture was stirred for 30 min to remove the starting materials and subsequently filtered; the isolated residue was washed with diethyl ether to obtain a yield for the desired complex of 43 mg (86%). Single crystals suitable for X-ray diffraction experiments were grown by slow vapor diffusion of *n*-hexane into an acetone solution of **II**. FTIR *ν*_CO_ (KBr pellet): 1916 (*s*), 1838 (*s*) cm^−1^.

## Refinement   

Crystal data, data collection, and structure refinement details are summarized in Table 3 ▸. All hydrogen atoms were placed at calculated positions (C—H = 0.95 Å) and refined using a riding model with *U*_iso_(H) = 1.2*U*_eq_(C). In compound **II**, the CO group and the Br atom *trans* to it were refined as being disordered over two sets of sites, (Br1/C2≡O2) and (Br2/C3≡O3), respectively, with an occupancy ratio of 0.807 (2): 0.193 (2).

## Supplementary Material

Crystal structure: contains datablock(s) global, I, II. DOI: 10.1107/S2056989020008178/wm5569sup1.cif

Structure factors: contains datablock(s) I. DOI: 10.1107/S2056989020008178/wm5569Isup2.hkl

Structure factors: contains datablock(s) II. DOI: 10.1107/S2056989020008178/wm5569IIsup3.hkl

Supporting information file. DOI: 10.1107/S2056989020008178/wm5569IIsup4.mol

CCDC references: 2010792, 2010791

Additional supporting information:  crystallographic information; 3D view; checkCIF report

## Figures and Tables

**Figure 2:**
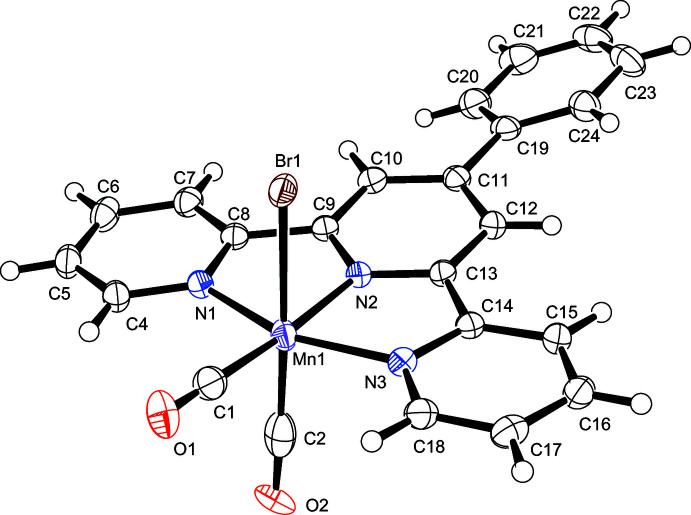
The mol­ecular structure of compound **II**, with atom labeling and displacement ellipsoids drawn at the 50% probability level. Only the major components (Br1/C2≡O2) of the disordered groups are shown.

**Table 1:** Hydrogen-bond geometry (Å, °) for **I** [Chem scheme1]

*D*—H⋯*A*	*D*—H	H⋯*A*	*D*⋯*A*	*D*—H⋯*A*
C7—H4⋯Br1^i^	0.95	2.83	3.754 (3)	165
C16—H8⋯Br1^ii^	0.95	2.88	3.612 (4)	135
C20—H11⋯Br1^i^	0.95	2.92	3.844 (2)	163

Symmetry codes: (i) 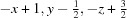; (ii) 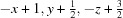.

**Table 2:** Hydrogen-bond geometry (Å, °) for **II** [Chem scheme1]

*D*—H⋯*A*	*D*—H	H⋯*A*	*D*⋯*A*	*D*—H⋯*A*
C5—H2⋯Br1^i^	0.95	2.84	3.528 (4)	130
C7—H4⋯Br1^ii^	0.95	2.86	3.771 (4)	162
C12—H6⋯Br2^iii^	0.95	2.75	3.688 (7)	171
C12—H6⋯O2^iii^	0.95	2.55	3.491 (7)	173
C15—H7⋯Br2^iii^	0.95	2.81	3.759 (7)	175
C15—H7⋯O2^iii^	0.95	2.50	3.447 (7)	172
C16—H8⋯Br2^iv^	0.95	2.52	3.286 (7)	138
C16—H8⋯O2^iv^	0.95	2.57	3.363 (7)	141
C20—H11⋯Br1^ii^	0.95	2.81	3.743 (4)	168
C20—H11⋯O3^ii^	0.95	2.55	3.446 (18)	158
C24—H15⋯Br2^iii^	0.95	2.84	3.611 (7)	139

Symmetry codes: (i) 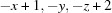; (ii) 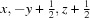; (iii) 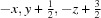; (iv) 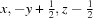.

**Table 3:** Experimental details

	**I**	**II**
Crystal data		
Chemical formula	[MnBr(C_21_H_15_N_3_)(CO)_3_]	[MnBr(C_21_H_15_N_3_)(CO)_2_]
*M*_r_	528.24	500.23
Crystal system, space group	Monoclinic, *P*2_1_/*c*	Monoclinic, *P*2_1_/*c*
Temperature (K)	93	93
*a*, *b*, *c* (Å)	11.6630 (3), 11.6691 (3), 15.8892 (4)	10.497 (3), 14.123 (5), 13.504 (4)
β (°)	103.0774 (7)	96.767 (3)
*V* (Å^3^)	2106.39 (10)	1988.0 (11)
*Z*	4	4
Radiation type	Mo *K*α	Mo *K*α
μ (mm^−1^)	2.56	2.71
Crystal size (mm)	0.15 × 0.08 × 0.03	0.20 × 0.08 × 0.05

Data collection		
Diffractometer	Rigaku Saturn70	Rigaku Saturn70
Absorption correction	Multi-scan (*REQAB*, Rigaku, 1998 ▸)	Multi-scan (*REQAB*, Rigaku, 1998 ▸)
*T*_min_, *T*_max_	0.774, 0.926	0.795, 0.873
No. of measured, independent and observed [*F* ^2^ > 2.0σ(*F* ^2^)] reflections	21455, 4813, 4253	19872, 4518, 4016
*R*_int_	0.030	0.028
(sin θ/λ)_max_ (Å^−1^)	0.649	0.649

Refinement		
*R*[*F* ^2^ > 2σ(*F* ^2^)], *wR*(*F* ^2^), *S*	0.035, 0.092, 1.06	0.046, 0.096, 1.27
No. of reflections	4813	4518
No. of parameters	289	289
No. of restraints	0	3
H-atom treatment	H-atom parameters constrained	H-atom parameters constrained
Δρ_max_, Δρ_min_ (e Å^−3^)	0.96, −0.32	0.83, −0.80

Computer programs: *PROCESS-AUTO* (Rigaku, 1998 ▸), *CrystalClear* (Rigaku, 2008 ▸), *SIR97* (Altomare *et al.*, 1999 ▸), *SHELXL2018/3* (Sheldrick, 2015 ▸), *Mercury* (Macrae *et al.*, 2020 ▸), *ORTEP-3 for Windows* (Farrugia, 2012 ▸), *CrystalStructure* (Rigaku, 2019 ▸), *PLATON* (Spek, 2020 ▸) and *publCIF* (Westrip, 2010 ▸).

## supplementary crystallographic information

### fac-Bromidotricarbonyl(4'-phenyl-2,2':6',2''-terpyridine-κ2N,N')manganese(I) (I) Crystal data

**Table:**

[MnBr(C_21_H_15_N_3_)(CO)_3_]	*F*(000) = 1056.00
*M**_r_* = 528.24	*D*_x_ = 1.666 Mg m^−^^3^
Monoclinic, *P*2_1_/*c*	Mo *K*α radiation, λ = 0.71075 Å
*a* = 11.6630 (3) Å	Cell parameters from 18973 reflections
*b* = 11.6691 (3) Å	θ = 3.0–27.5°
*c* = 15.8892 (4) Å	µ = 2.56 mm^−^^1^
β = 103.0774 (7)°	*T* = 93 K
*V* = 2106.39 (10) Å^3^	Platelet, orange
*Z* = 4	0.15 × 0.08 × 0.03 mm

### fac-Bromidotricarbonyl(4'-phenyl-2,2':6',2''-terpyridine-κ2N,N')manganese(I) (I) Data collection

**Table:**

Rigaku Saturn70 diffractometer	4253 reflections with *F*^2^ > 2.0σ(*F*^2^)
Detector resolution: 7.143 pixels mm^-1^	*R*_int_ = 0.030
ω scans	θ_max_ = 27.5°, θ_min_ = 3.0°
Absorption correction: multi-scan (*REQAB*, Rigaku, 1998)	*h* = −15→15
*T*_min_ = 0.774, *T*_max_ = 0.926	*k* = −15→15
21455 measured reflections	*l* = −20→19
4813 independent reflections	

### fac-Bromidotricarbonyl(4'-phenyl-2,2':6',2''-terpyridine-κ2N,N')manganese(I) (I) Refinement

**Table:**

Refinement on *F*^2^	Secondary atom site location: difference Fourier map
*R*[*F*^2^ > 2σ(*F*^2^)] = 0.035	Hydrogen site location: inferred from neighbouring sites
*wR*(*F*^2^) = 0.092	H-atom parameters constrained
*S* = 1.06	*w* = 1/[σ^2^(*F*_o_^2^) + (0.0416*P*)^2^ + 2.6282*P*] where *P* = (*F*_o_^2^ + 2*F*_c_^2^)/3
4813 reflections	(Δ/σ)_max_ = 0.001
289 parameters	Δρ_max_ = 0.96 e Å^−^^3^
0 restraints	Δρ_min_ = −0.32 e Å^−^^3^
Primary atom site location: structure-invariant direct methods	

### fac-Bromidotricarbonyl(4'-phenyl-2,2':6',2''-terpyridine-κ2N,N')manganese(I) (I) Special details

**Table:**

Refinement. Refinement was performed using all reflections. The weighted R-factor (wR) and goodness of fit (S) are based on F^2^. R-factor (gt) are based on F. The threshold expression of F^2^ > 2.0 sigma(F^2^) is used only for calculating R-factor (gt).

### fac-Bromidotricarbonyl(4'-phenyl-2,2':6',2''-terpyridine-κ2N,N')manganese(I) (I) Fractional atomic coordinates and isotropic or equivalent isotropic displacement parameters (Å^2^)

**Table:**

	*x*	*y*	*z*	*U*_iso_*/*U*_eq_	
Br1	0.61476 (2)	0.17999 (2)	0.88742 (2)	0.02812 (9)	
Mn1	0.78248 (3)	0.17015 (3)	0.81054 (2)	0.02513 (10)	
O1	0.99149 (17)	0.17222 (19)	0.73649 (14)	0.0400 (5)	
O2	0.7925 (2)	0.42515 (18)	0.81993 (14)	0.0444 (5)	
O3	0.95375 (19)	0.1798 (2)	0.97692 (14)	0.0457 (5)	
N1	0.76212 (17)	−0.00325 (19)	0.81015 (13)	0.0252 (4)	
N2	0.65411 (17)	0.14582 (19)	0.69692 (13)	0.0238 (4)	
N3	0.7794 (2)	0.3305 (2)	0.62983 (17)	0.0359 (5)	
C1	0.9075 (2)	0.1690 (2)	0.76117 (18)	0.0320 (6)	
C2	0.7848 (3)	0.3278 (3)	0.81319 (19)	0.0354 (6)	
C3	0.8852 (2)	0.1758 (2)	0.91343 (18)	0.0327 (6)	
C4	0.8291 (2)	−0.0776 (2)	0.86473 (17)	0.0313 (6)	
H1	0.893046	−0.048384	0.907298	0.038*	
C5	0.8095 (2)	−0.1941 (3)	0.86198 (19)	0.0358 (6)	
H2	0.860275	−0.243958	0.900658	0.043*	
C6	0.7144 (2)	−0.2373 (3)	0.80188 (19)	0.0350 (6)	
H3	0.698453	−0.317169	0.798723	0.042*	
C7	0.6431 (2)	−0.1615 (2)	0.74643 (18)	0.0313 (6)	
H4	0.576614	−0.188952	0.705236	0.038*	
C8	0.6693 (2)	−0.0454 (2)	0.75134 (15)	0.0247 (5)	
C9	0.6038 (2)	0.0406 (2)	0.69157 (15)	0.0232 (5)	
C10	0.4996 (2)	0.0143 (2)	0.63271 (15)	0.0232 (5)	
H5	0.466483	−0.060220	0.632112	0.028*	
C11	0.44359 (19)	0.0971 (2)	0.57461 (15)	0.0214 (5)	
C12	0.5003 (2)	0.2023 (2)	0.57681 (16)	0.0248 (5)	
H6	0.467288	0.260028	0.536409	0.030*	
C13	0.6042 (2)	0.2244 (2)	0.63698 (16)	0.0254 (5)	
C14	0.6644 (2)	0.3362 (2)	0.63063 (17)	0.0280 (5)	
C15	0.6033 (2)	0.4378 (2)	0.62349 (19)	0.0326 (6)	
H7	0.522062	0.438538	0.624227	0.039*	
C16	0.6619 (3)	0.5393 (3)	0.6152 (2)	0.0418 (7)	
H8	0.621545	0.610530	0.610537	0.050*	
C17	0.7798 (3)	0.5349 (3)	0.6138 (2)	0.0446 (7)	
H9	0.822144	0.602872	0.607979	0.054*	
C18	0.8345 (3)	0.4297 (3)	0.6212 (2)	0.0403 (7)	
H10	0.915543	0.426919	0.620071	0.048*	
C19	0.3301 (2)	0.0749 (2)	0.51219 (15)	0.0215 (5)	
C20	0.2826 (2)	−0.0347 (2)	0.49944 (17)	0.0316 (6)	
H11	0.324220	−0.097276	0.530401	0.038*	
C21	0.1756 (3)	−0.0542 (3)	0.44231 (19)	0.0389 (7)	
H12	0.144475	−0.129640	0.434142	0.047*	
C22	0.1145 (2)	0.0361 (3)	0.39740 (19)	0.0373 (6)	
H13	0.039539	0.023966	0.359983	0.045*	
C23	0.1626 (3)	0.1429 (3)	0.4072 (2)	0.0453 (8)	
H14	0.122516	0.204709	0.374184	0.054*	
C24	0.2688 (3)	0.1626 (2)	0.4644 (2)	0.0402 (7)	
H15	0.300073	0.238042	0.470790	0.048*	

### fac-Bromidotricarbonyl(4'-phenyl-2,2':6',2''-terpyridine-κ2N,N')manganese(I) (I) Atomic displacement parameters (Å^2^)

**Table:**

	*U*^11^	*U*^22^	*U*^33^	*U*^12^	*U*^13^	*U*^23^
Br1	0.02474 (13)	0.03228 (14)	0.02610 (14)	0.00130 (9)	0.00315 (10)	−0.00370 (10)
Mn1	0.01744 (18)	0.0327 (2)	0.0221 (2)	−0.00087 (14)	−0.00207 (14)	−0.00159 (15)
O1	0.0209 (9)	0.0603 (14)	0.0372 (11)	−0.0046 (9)	0.0033 (8)	−0.0054 (10)
O2	0.0517 (13)	0.0352 (11)	0.0424 (12)	−0.0087 (10)	0.0023 (10)	−0.0043 (9)
O3	0.0325 (11)	0.0571 (14)	0.0377 (12)	−0.0050 (9)	−0.0125 (9)	−0.0002 (10)
N1	0.0163 (9)	0.0359 (11)	0.0211 (10)	0.0032 (8)	−0.0007 (8)	−0.0008 (8)
N2	0.0168 (9)	0.0321 (11)	0.0209 (10)	0.0015 (8)	0.0005 (8)	−0.0036 (8)
N3	0.0263 (11)	0.0384 (13)	0.0427 (14)	−0.0042 (9)	0.0072 (10)	−0.0009 (10)
C1	0.0241 (13)	0.0399 (15)	0.0271 (13)	−0.0049 (10)	−0.0045 (10)	−0.0031 (11)
C2	0.0300 (14)	0.0417 (16)	0.0323 (15)	−0.0015 (11)	0.0028 (11)	−0.0017 (12)
C3	0.0303 (14)	0.0343 (14)	0.0312 (14)	−0.0039 (11)	0.0019 (11)	−0.0009 (11)
C4	0.0212 (12)	0.0407 (15)	0.0271 (13)	0.0043 (10)	−0.0049 (10)	−0.0010 (11)
C5	0.0266 (13)	0.0432 (16)	0.0323 (15)	0.0071 (11)	−0.0041 (11)	0.0091 (12)
C6	0.0300 (13)	0.0368 (15)	0.0345 (15)	−0.0005 (11)	−0.0005 (11)	0.0060 (12)
C7	0.0252 (12)	0.0385 (15)	0.0266 (13)	−0.0036 (10)	−0.0017 (10)	0.0053 (11)
C8	0.0178 (11)	0.0354 (13)	0.0198 (11)	0.0014 (9)	0.0019 (9)	0.0013 (10)
C9	0.0178 (10)	0.0328 (13)	0.0186 (11)	0.0010 (9)	0.0031 (9)	0.0008 (9)
C10	0.0187 (10)	0.0280 (12)	0.0212 (11)	−0.0022 (9)	0.0009 (9)	0.0020 (9)
C11	0.0165 (10)	0.0282 (12)	0.0184 (11)	0.0000 (9)	0.0016 (8)	−0.0003 (9)
C12	0.0185 (11)	0.0276 (12)	0.0263 (12)	0.0017 (9)	0.0007 (9)	0.0011 (10)
C13	0.0196 (11)	0.0287 (12)	0.0267 (13)	0.0008 (9)	0.0027 (10)	−0.0034 (10)
C14	0.0246 (12)	0.0308 (13)	0.0276 (13)	−0.0025 (10)	0.0038 (10)	−0.0005 (10)
C15	0.0259 (12)	0.0313 (13)	0.0412 (15)	−0.0036 (10)	0.0089 (11)	−0.0030 (11)
C16	0.0374 (15)	0.0309 (14)	0.0568 (19)	−0.0007 (12)	0.0103 (14)	0.0005 (13)
C17	0.0380 (16)	0.0354 (15)	0.060 (2)	−0.0105 (13)	0.0105 (14)	0.0009 (14)
C18	0.0279 (14)	0.0428 (16)	0.0506 (18)	−0.0074 (12)	0.0100 (13)	−0.0054 (14)
C19	0.0179 (10)	0.0283 (12)	0.0172 (11)	0.0023 (9)	0.0018 (9)	−0.0002 (9)
C20	0.0300 (13)	0.0320 (13)	0.0278 (13)	−0.0007 (10)	−0.0039 (11)	0.0056 (11)
C21	0.0344 (15)	0.0347 (15)	0.0399 (16)	−0.0088 (12)	−0.0078 (12)	0.0015 (12)
C22	0.0246 (13)	0.0417 (16)	0.0368 (15)	0.0031 (11)	−0.0115 (11)	−0.0062 (12)
C23	0.0420 (17)	0.0332 (15)	0.0465 (18)	0.0079 (13)	−0.0194 (14)	0.0018 (13)
C24	0.0377 (16)	0.0266 (13)	0.0432 (17)	−0.0041 (11)	−0.0182 (13)	0.0049 (12)

### fac-Bromidotricarbonyl(4'-phenyl-2,2':6',2''-terpyridine-κ2N,N')manganese(I) (I) Geometric parameters (Å, º)

**Table:**

Br1—Mn1	2.5325 (5)	C10—H5	0.9500
Mn1—C3	1.796 (3)	C11—C12	1.390 (3)
Mn1—C1	1.805 (3)	C11—C19	1.486 (3)
Mn1—C2	1.840 (3)	C12—C13	1.388 (3)
Mn1—N1	2.037 (2)	C12—H6	0.9500
Mn1—N2	2.088 (2)	C13—C14	1.496 (3)
O1—C1	1.135 (4)	C14—C15	1.375 (4)
O2—C2	1.143 (3)	C15—C16	1.388 (4)
O3—C3	1.138 (3)	C15—H7	0.9500
N1—C4	1.345 (3)	C16—C17	1.381 (4)
N1—C8	1.353 (3)	C16—H8	0.9500
N2—C9	1.355 (3)	C17—C18	1.376 (4)
N2—C13	1.355 (3)	C17—H9	0.9500
N3—C18	1.346 (4)	C18—H10	0.9500
N3—C14	1.347 (3)	C19—C24	1.374 (3)
C4—C5	1.377 (4)	C19—C20	1.391 (4)
C4—H1	0.9500	C20—C21	1.386 (4)
C5—C6	1.384 (4)	C20—H11	0.9500
C5—H2	0.9500	C21—C22	1.377 (4)
C6—C7	1.385 (4)	C21—H12	0.9500
C6—H3	0.9500	C22—C23	1.361 (4)
C7—C8	1.387 (4)	C22—H13	0.9500
C7—H4	0.9500	C23—C24	1.379 (4)
C8—C9	1.471 (3)	C23—H14	0.9500
C9—C10	1.390 (3)	C24—H15	0.9500
C10—C11	1.393 (3)		
			
C3—Mn1—C1	87.58 (13)	C9—C10—H5	120.0
C3—Mn1—C2	86.53 (13)	C11—C10—H5	120.0
C1—Mn1—C2	90.48 (13)	C12—C11—C10	116.6 (2)
C3—Mn1—N1	95.30 (10)	C12—C11—C19	121.2 (2)
C1—Mn1—N1	95.53 (11)	C10—C11—C19	122.3 (2)
C2—Mn1—N1	173.78 (11)	C13—C12—C11	121.2 (2)
C3—Mn1—N2	172.86 (11)	C13—C12—H6	119.4
C1—Mn1—N2	96.60 (10)	C11—C12—H6	119.4
C2—Mn1—N2	99.18 (11)	N2—C13—C12	121.9 (2)
N1—Mn1—N2	78.58 (8)	N2—C13—C14	120.3 (2)
C3—Mn1—Br1	89.33 (9)	C12—C13—C14	117.7 (2)
C1—Mn1—Br1	176.28 (9)	N3—C14—C15	122.7 (2)
C2—Mn1—Br1	87.27 (9)	N3—C14—C13	116.2 (2)
N1—Mn1—Br1	86.80 (6)	C15—C14—C13	121.0 (2)
N2—Mn1—Br1	86.69 (6)	C14—C15—C16	119.1 (3)
C4—N1—C8	117.9 (2)	C14—C15—H7	120.4
C4—N1—Mn1	126.07 (17)	C16—C15—H7	120.4
C8—N1—Mn1	115.98 (16)	C17—C16—C15	118.9 (3)
C9—N2—C13	117.3 (2)	C17—C16—H8	120.6
C9—N2—Mn1	113.14 (16)	C15—C16—H8	120.6
C13—N2—Mn1	128.74 (17)	C18—C17—C16	118.4 (3)
C18—N3—C14	117.3 (2)	C18—C17—H9	120.8
O1—C1—Mn1	174.2 (2)	C16—C17—H9	120.8
O2—C2—Mn1	175.2 (3)	N3—C18—C17	123.6 (3)
O3—C3—Mn1	177.3 (3)	N3—C18—H10	118.2
N1—C4—C5	123.3 (2)	C17—C18—H10	118.2
N1—C4—H1	118.4	C24—C19—C20	117.6 (2)
C5—C4—H1	118.4	C24—C19—C11	120.9 (2)
C4—C5—C6	118.8 (3)	C20—C19—C11	121.5 (2)
C4—C5—H2	120.6	C21—C20—C19	121.1 (2)
C6—C5—H2	120.6	C21—C20—H11	119.5
C7—C6—C5	118.6 (3)	C19—C20—H11	119.5
C7—C6—H3	120.7	C22—C21—C20	119.8 (3)
C5—C6—H3	120.7	C22—C21—H12	120.1
C6—C7—C8	119.7 (2)	C20—C21—H12	120.1
C6—C7—H4	120.2	C23—C22—C21	119.3 (2)
C8—C7—H4	120.2	C23—C22—H13	120.3
N1—C8—C7	121.7 (2)	C21—C22—H13	120.3
N1—C8—C9	114.5 (2)	C22—C23—C24	120.9 (3)
C7—C8—C9	123.7 (2)	C22—C23—H14	119.6
N2—C9—C10	122.9 (2)	C24—C23—H14	119.6
N2—C9—C8	115.1 (2)	C19—C24—C23	121.2 (3)
C10—C9—C8	122.0 (2)	C19—C24—H15	119.4
C9—C10—C11	120.0 (2)	C23—C24—H15	119.4
			
C8—N1—C4—C5	1.8 (4)	Mn1—N2—C13—C14	20.3 (3)
Mn1—N1—C4—C5	178.9 (2)	C11—C12—C13—N2	−1.1 (4)
N1—C4—C5—C6	−1.9 (5)	C11—C12—C13—C14	175.4 (2)
C4—C5—C6—C7	0.4 (4)	C18—N3—C14—C15	−0.3 (4)
C5—C6—C7—C8	1.0 (4)	C18—N3—C14—C13	177.8 (3)
C4—N1—C8—C7	−0.3 (4)	N2—C13—C14—N3	47.9 (3)
Mn1—N1—C8—C7	−177.7 (2)	C12—C13—C14—N3	−128.7 (3)
C4—N1—C8—C9	−177.3 (2)	N2—C13—C14—C15	−134.0 (3)
Mn1—N1—C8—C9	5.3 (3)	C12—C13—C14—C15	49.5 (4)
C6—C7—C8—N1	−1.1 (4)	N3—C14—C15—C16	−0.1 (4)
C6—C7—C8—C9	175.7 (2)	C13—C14—C15—C16	−178.1 (3)
C13—N2—C9—C10	−5.6 (3)	C14—C15—C16—C17	0.4 (5)
Mn1—N2—C9—C10	164.82 (19)	C15—C16—C17—C18	−0.3 (5)
C13—N2—C9—C8	173.0 (2)	C14—N3—C18—C17	0.5 (5)
Mn1—N2—C9—C8	−16.7 (3)	C16—C17—C18—N3	−0.2 (5)
N1—C8—C9—N2	7.9 (3)	C12—C11—C19—C24	−10.3 (4)
C7—C8—C9—N2	−169.1 (2)	C10—C11—C19—C24	170.7 (3)
N1—C8—C9—C10	−173.6 (2)	C12—C11—C19—C20	169.1 (2)
C7—C8—C9—C10	9.4 (4)	C10—C11—C19—C20	−9.9 (4)
N2—C9—C10—C11	1.4 (4)	C24—C19—C20—C21	−1.8 (4)
C8—C9—C10—C11	−177.0 (2)	C11—C19—C20—C21	178.7 (3)
C9—C10—C11—C12	2.9 (3)	C19—C20—C21—C22	−0.2 (5)
C9—C10—C11—C19	−178.0 (2)	C20—C21—C22—C23	2.7 (5)
C10—C11—C12—C13	−3.1 (4)	C21—C22—C23—C24	−3.1 (5)
C19—C11—C12—C13	177.8 (2)	C20—C19—C24—C23	1.4 (5)
C9—N2—C13—C12	5.3 (3)	C11—C19—C24—C23	−179.1 (3)
Mn1—N2—C13—C12	−163.30 (18)	C22—C23—C24—C19	1.0 (6)
C9—N2—C13—C14	−171.0 (2)		

### fac-Bromidotricarbonyl(4'-phenyl-2,2':6',2''-terpyridine-κ2N,N')manganese(I) (I) Hydrogen-bond geometry (Å, º)

**Table:**

*D*—H···*A*	*D*—H	H···*A*	*D*···*A*	*D*—H···*A*
C7—H4···Br1^i^	0.95	2.83	3.754 (3)	165
C16—H8···Br1^ii^	0.95	2.88	3.612 (4)	135
C20—H11···Br1^i^	0.95	2.92	3.844 (2)	163

Symmetry codes: (i) −*x*+1, *y*−1/2, −*z*+3/2; (ii) −*x*+1, *y*+1/2, −*z*+3/2.

### cis-Bromidodicarbonyl(4'-phenyl-2,2':6',2''-terpyridine-κ3N,N',N'')manganese(I) (II) Crystal data

**Table:**

[MnBr(C_21_H_15_N_3_)(CO)_2_]	*F*(000) = 1000.00
*M**_r_* = 500.23	*D*_x_ = 1.671 Mg m^−^^3^
Monoclinic, *P*2_1_/*c*	Mo *K*α radiation, λ = 0.71075 Å
*a* = 10.497 (3) Å	Cell parameters from 5160 reflections
*b* = 14.123 (5) Å	θ = 3.0–27.5°
*c* = 13.504 (4) Å	µ = 2.71 mm^−^^1^
β = 96.767 (3)°	*T* = 93 K
*V* = 1988.0 (11) Å^3^	Block, red
*Z* = 4	0.20 × 0.08 × 0.05 mm

### cis-Bromidodicarbonyl(4'-phenyl-2,2':6',2''-terpyridine-κ3N,N',N'')manganese(I) (II) Data collection

**Table:**

Rigaku Saturn70 diffractometer	4016 reflections with *F*^2^ > 2.0σ(*F*^2^)
Detector resolution: 28.626 pixels mm^-1^	*R*_int_ = 0.028
ω scans	θ_max_ = 27.5°, θ_min_ = 3.0°
Absorption correction: multi-scan (*REQAB*, Rigaku, 1998)	*h* = −13→13
*T*_min_ = 0.795, *T*_max_ = 0.873	*k* = −18→18
19872 measured reflections	*l* = −17→17
4518 independent reflections	

### cis-Bromidodicarbonyl(4'-phenyl-2,2':6',2''-terpyridine-κ3N,N',N'')manganese(I) (II) Refinement

**Table:**

Refinement on *F*^2^	Secondary atom site location: difference Fourier map
*R*[*F*^2^ > 2σ(*F*^2^)] = 0.046	Hydrogen site location: inferred from neighbouring sites
*wR*(*F*^2^) = 0.096	H-atom parameters constrained
*S* = 1.27	*w* = 1/[σ^2^(*F*_o_^2^) + (0.0039*P*)^2^ + 5.6867*P*] where *P* = (*F*_o_^2^ + 2*F*_c_^2^)/3
4518 reflections	(Δ/σ)_max_ < 0.001
289 parameters	Δρ_max_ = 0.83 e Å^−^^3^
3 restraints	Δρ_min_ = −0.80 e Å^−^^3^
Primary atom site location: structure-invariant direct methods	

### cis-Bromidodicarbonyl(4'-phenyl-2,2':6',2''-terpyridine-κ3N,N',N'')manganese(I) (II) Special details

**Table:**

Refinement. Refinement was performed using all reflections. The weighted R-factor (wR) and goodness of fit (S) are based on F^2^. R-factor (gt) are based on F. The threshold expression of F^2^ > 2.0 sigma(F^2^) is used only for calculating R-factor (gt).

### cis-Bromidodicarbonyl(4'-phenyl-2,2':6',2''-terpyridine-κ3N,N',N'')manganese(I) (II) Fractional atomic coordinates and isotropic or equivalent isotropic displacement parameters (Å^2^)

**Table:**

	*x*	*y*	*z*	*U*_iso_*/*U*_eq_	Occ. (<1)
Br1	0.39194 (6)	0.11344 (3)	0.73710 (4)	0.02535 (17)	0.807 (2)
O2	−0.0653 (5)	0.0445 (4)	0.8859 (4)	0.0351 (15)	0.807 (2)
C2	0.0332 (9)	0.0641 (9)	0.8536 (10)	0.037 (3)	0.807 (2)
Br2	−0.0197 (5)	0.0661 (4)	0.8657 (4)	0.0403 (14)	0.193 (2)
O3	0.4377 (18)	0.1006 (12)	0.7111 (13)	0.033 (4)*	0.193 (2)
C3	0.339 (3)	0.092 (3)	0.746 (3)	0.076 (12)*	0.193 (2)
Mn1	0.18158 (5)	0.08786 (4)	0.80533 (4)	0.02451 (14)	
O1	0.1797 (3)	−0.10749 (19)	0.7376 (2)	0.0359 (6)	
N1	0.2796 (3)	0.0760 (2)	0.9421 (2)	0.0224 (6)	
N2	0.2005 (3)	0.22091 (19)	0.8440 (2)	0.0202 (6)	
N3	0.0937 (3)	0.1521 (2)	0.6822 (2)	0.0219 (6)	
C1	0.1807 (4)	−0.0334 (3)	0.7637 (3)	0.0310 (8)	
C4	0.3125 (3)	−0.0050 (3)	0.9917 (3)	0.0275 (8)	
H1	0.288826	−0.063547	0.960043	0.033*	
C5	0.3784 (4)	−0.0070 (3)	1.0856 (3)	0.0298 (8)	
H2	0.400366	−0.065698	1.117441	0.036*	
C6	0.4125 (4)	0.0773 (3)	1.1335 (3)	0.0301 (8)	
H3	0.458098	0.077281	1.198549	0.036*	
C7	0.3789 (3)	0.1623 (3)	1.0847 (3)	0.0257 (7)	
H4	0.400583	0.221221	1.116233	0.031*	
C8	0.3132 (3)	0.1592 (2)	0.9894 (3)	0.0217 (7)	
C9	0.2711 (3)	0.2443 (2)	0.9303 (3)	0.0212 (7)	
C10	0.3006 (3)	0.3376 (2)	0.9548 (3)	0.0227 (7)	
H5	0.351261	0.352547	1.015754	0.027*	
C11	0.2548 (3)	0.4096 (2)	0.8886 (3)	0.0223 (7)	
C12	0.1818 (3)	0.3838 (2)	0.7994 (3)	0.0225 (7)	
H6	0.149023	0.431227	0.753384	0.027*	
C13	0.1572 (3)	0.2887 (2)	0.7779 (3)	0.0208 (7)	
C14	0.0918 (3)	0.2483 (2)	0.6854 (3)	0.0210 (7)	
C15	0.0335 (3)	0.3016 (3)	0.6061 (3)	0.0251 (7)	
H7	0.033795	0.368774	0.609582	0.030*	
C16	−0.0251 (3)	0.2560 (3)	0.5220 (3)	0.0274 (8)	
H8	−0.066553	0.291366	0.467603	0.033*	
C17	−0.0223 (3)	0.1587 (3)	0.5187 (3)	0.0277 (8)	
H9	−0.060962	0.125743	0.461531	0.033*	
C18	0.0376 (3)	0.1095 (3)	0.5994 (3)	0.0262 (7)	
H10	0.039210	0.042307	0.596314	0.031*	
C19	0.2863 (3)	0.5109 (2)	0.9128 (3)	0.0247 (7)	
C20	0.3268 (4)	0.5390 (3)	1.0100 (3)	0.0316 (8)	
H11	0.333688	0.493598	1.062254	0.038*	
C21	0.3573 (4)	0.6332 (3)	1.0315 (3)	0.0385 (10)	
H12	0.385755	0.651422	1.098118	0.046*	
C22	0.3466 (4)	0.7000 (3)	0.9572 (4)	0.0379 (10)	
H13	0.367454	0.764157	0.972651	0.045*	
C23	0.3056 (4)	0.6739 (3)	0.8598 (3)	0.0376 (10)	
H14	0.297827	0.720240	0.808386	0.045*	
C24	0.2757 (4)	0.5797 (3)	0.8373 (3)	0.0299 (8)	
H15	0.247979	0.561819	0.770438	0.036*	

### cis-Bromidodicarbonyl(4'-phenyl-2,2':6',2''-terpyridine-κ3N,N',N'')manganese(I) (II) Atomic displacement parameters (Å^2^)

**Table:**

	*U*^11^	*U*^22^	*U*^33^	*U*^12^	*U*^13^	*U*^23^
Br1	0.0222 (3)	0.0293 (3)	0.0246 (3)	0.0054 (2)	0.0032 (2)	0.00736 (19)
O2	0.038 (3)	0.028 (3)	0.041 (3)	−0.0170 (18)	0.012 (2)	0.000 (2)
C2	0.055 (7)	0.023 (3)	0.031 (4)	0.009 (5)	−0.006 (5)	−0.006 (3)
Br2	0.060 (4)	0.0228 (17)	0.035 (2)	0.001 (3)	−0.005 (3)	−0.0077 (14)
Mn1	0.0276 (3)	0.0170 (3)	0.0275 (3)	0.0001 (2)	−0.0027 (2)	0.0030 (2)
O1	0.0504 (18)	0.0244 (14)	0.0342 (15)	0.0084 (12)	0.0102 (13)	0.0043 (12)
N1	0.0202 (14)	0.0197 (14)	0.0276 (15)	0.0010 (11)	0.0042 (12)	0.0048 (12)
N2	0.0176 (13)	0.0179 (13)	0.0250 (15)	0.0001 (10)	0.0017 (11)	0.0029 (11)
N3	0.0191 (14)	0.0215 (14)	0.0249 (15)	0.0006 (11)	0.0016 (12)	0.0015 (12)
C1	0.0266 (19)	0.037 (2)	0.029 (2)	−0.0021 (16)	0.0024 (15)	0.0106 (17)
C4	0.0248 (18)	0.0231 (17)	0.035 (2)	0.0036 (14)	0.0064 (15)	0.0077 (15)
C5	0.0276 (19)	0.0283 (19)	0.034 (2)	0.0070 (15)	0.0064 (16)	0.0115 (16)
C6	0.0286 (19)	0.039 (2)	0.0235 (18)	0.0076 (16)	0.0040 (15)	0.0082 (16)
C7	0.0239 (17)	0.0283 (18)	0.0252 (18)	0.0022 (14)	0.0041 (14)	0.0017 (14)
C8	0.0182 (16)	0.0240 (17)	0.0236 (17)	0.0012 (13)	0.0059 (13)	0.0031 (14)
C9	0.0177 (16)	0.0214 (16)	0.0249 (17)	0.0006 (13)	0.0042 (13)	0.0030 (13)
C10	0.0192 (16)	0.0247 (17)	0.0244 (18)	0.0003 (13)	0.0028 (13)	−0.0012 (14)
C11	0.0180 (16)	0.0202 (16)	0.0297 (18)	0.0012 (12)	0.0064 (14)	0.0008 (14)
C12	0.0208 (16)	0.0196 (16)	0.0270 (18)	0.0014 (13)	0.0028 (13)	0.0027 (14)
C13	0.0172 (15)	0.0224 (17)	0.0232 (17)	0.0012 (12)	0.0036 (13)	0.0041 (13)
C14	0.0173 (15)	0.0209 (16)	0.0251 (17)	0.0000 (12)	0.0037 (13)	0.0018 (13)
C15	0.0221 (17)	0.0243 (17)	0.0289 (19)	0.0016 (14)	0.0023 (14)	0.0057 (14)
C16	0.0225 (17)	0.0333 (19)	0.0261 (19)	0.0011 (15)	0.0016 (14)	0.0059 (15)
C17	0.0227 (18)	0.036 (2)	0.0241 (18)	−0.0009 (15)	0.0013 (14)	−0.0016 (15)
C18	0.0224 (17)	0.0247 (17)	0.0311 (19)	−0.0016 (14)	0.0020 (14)	−0.0005 (15)
C19	0.0181 (16)	0.0208 (17)	0.036 (2)	−0.0005 (13)	0.0065 (14)	−0.0015 (14)
C20	0.0290 (19)	0.0240 (18)	0.042 (2)	0.0000 (15)	0.0027 (17)	−0.0041 (16)
C21	0.031 (2)	0.031 (2)	0.053 (3)	−0.0024 (16)	0.0019 (19)	−0.0145 (19)
C22	0.030 (2)	0.0206 (18)	0.065 (3)	−0.0047 (15)	0.014 (2)	−0.0097 (19)
C23	0.038 (2)	0.0211 (18)	0.057 (3)	−0.0022 (16)	0.018 (2)	0.0041 (18)
C24	0.0288 (19)	0.0221 (17)	0.040 (2)	0.0013 (14)	0.0081 (16)	0.0006 (16)

### cis-Bromidodicarbonyl(4'-phenyl-2,2':6',2''-terpyridine-κ3N,N',N'')manganese(I) (II) Geometric parameters (Å, º)

**Table:**

Br1—O3	0.65 (2)	C9—C10	1.384 (5)
Br1—C3	0.66 (3)	C10—C11	1.401 (5)
Br1—Mn1	2.5170 (11)	C10—H5	0.9500
O2—Br2	0.654 (5)	C11—C12	1.398 (5)
O2—C2	1.201 (11)	C11—C19	1.496 (5)
C2—Br2	0.597 (8)	C12—C13	1.392 (5)
C2—Mn1	1.790 (9)	C12—H6	0.9500
O3—C3	1.194 (18)	C13—C14	1.468 (5)
Mn1—C1	1.803 (4)	C14—C15	1.390 (5)
Mn1—N2	1.954 (3)	C15—C16	1.384 (5)
Mn1—N1	2.012 (3)	C15—H7	0.9500
Mn1—N3	2.019 (3)	C16—C17	1.376 (5)
O1—C1	1.103 (5)	C16—H8	0.9500
N1—C4	1.350 (4)	C17—C18	1.380 (5)
N1—C8	1.364 (4)	C17—H9	0.9500
N2—C9	1.346 (4)	C18—H10	0.9500
N2—C13	1.351 (4)	C19—C20	1.390 (5)
N3—C18	1.343 (4)	C19—C24	1.402 (5)
N3—C14	1.360 (4)	C20—C21	1.390 (5)
C4—C5	1.371 (5)	C20—H11	0.9500
C4—H1	0.9500	C21—C22	1.371 (6)
C5—C6	1.381 (6)	C21—H12	0.9500
C5—H2	0.9500	C22—C23	1.385 (6)
C6—C7	1.395 (5)	C22—H13	0.9500
C6—H3	0.9500	C23—C24	1.393 (5)
C7—C8	1.387 (5)	C23—H14	0.9500
C7—H4	0.9500	C24—H15	0.9500
C8—C9	1.481 (5)		
			
O3—Br1—C3	131 (4)	N2—C9—C8	111.5 (3)
Br2—O2—C2	15.7 (10)	C10—C9—C8	126.8 (3)
Br2—C2—O2	17.2 (12)	C9—C10—C11	119.2 (3)
O2—C2—Mn1	177.5 (10)	C9—C10—H5	120.4
C2—Br2—O2	147 (2)	C11—C10—H5	120.4
Br1—O3—C3	24 (2)	C12—C11—C10	118.2 (3)
Br1—C3—O3	24.3 (19)	C12—C11—C19	121.5 (3)
C2—Mn1—C1	87.9 (4)	C10—C11—C19	120.3 (3)
C2—Mn1—N2	98.6 (4)	C13—C12—C11	120.0 (3)
C1—Mn1—N2	173.57 (15)	C13—C12—H6	120.0
C2—Mn1—N1	91.3 (4)	C11—C12—H6	120.0
C1—Mn1—N1	100.98 (14)	N2—C13—C12	120.4 (3)
N2—Mn1—N1	79.05 (12)	N2—C13—C14	112.0 (3)
C2—Mn1—N3	93.0 (4)	C12—C13—C14	127.5 (3)
C1—Mn1—N3	100.66 (15)	N3—C14—C15	121.5 (3)
N2—Mn1—N3	79.05 (12)	N3—C14—C13	114.1 (3)
N1—Mn1—N3	158.08 (12)	C15—C14—C13	124.4 (3)
C2—Mn1—Br1	177.4 (4)	C16—C15—C14	119.6 (3)
C1—Mn1—Br1	89.68 (12)	C16—C15—H7	120.2
N2—Mn1—Br1	83.89 (8)	C14—C15—H7	120.2
N1—Mn1—Br1	88.42 (8)	C17—C16—C15	118.8 (3)
N3—Mn1—Br1	88.26 (8)	C17—C16—H8	120.6
C4—N1—C8	117.4 (3)	C15—C16—H8	120.6
C4—N1—Mn1	126.8 (3)	C16—C17—C18	119.1 (3)
C8—N1—Mn1	115.8 (2)	C16—C17—H9	120.4
C9—N2—C13	120.4 (3)	C18—C17—H9	120.4
C9—N2—Mn1	119.7 (2)	N3—C18—C17	123.1 (3)
C13—N2—Mn1	119.3 (2)	N3—C18—H10	118.4
C18—N3—C14	117.9 (3)	C17—C18—H10	118.4
C18—N3—Mn1	126.7 (2)	C20—C19—C24	118.5 (3)
C14—N3—Mn1	115.5 (2)	C20—C19—C11	121.0 (3)
O1—C1—Mn1	179.5 (4)	C24—C19—C11	120.5 (3)
N1—C4—C5	123.2 (4)	C21—C20—C19	120.5 (4)
N1—C4—H1	118.4	C21—C20—H11	119.8
C5—C4—H1	118.4	C19—C20—H11	119.8
C4—C5—C6	119.4 (3)	C22—C21—C20	120.6 (4)
C4—C5—H2	120.3	C22—C21—H12	119.7
C6—C5—H2	120.3	C20—C21—H12	119.7
C5—C6—C7	118.9 (3)	C21—C22—C23	120.1 (4)
C5—C6—H3	120.5	C21—C22—H13	120.0
C7—C6—H3	120.5	C23—C22—H13	120.0
C8—C7—C6	118.8 (3)	C22—C23—C24	119.8 (4)
C8—C7—H4	120.6	C22—C23—H14	120.1
C6—C7—H4	120.6	C24—C23—H14	120.1
N1—C8—C7	122.3 (3)	C23—C24—C19	120.5 (4)
N1—C8—C9	113.7 (3)	C23—C24—H15	119.8
C7—C8—C9	124.0 (3)	C19—C24—H15	119.8
N2—C9—C10	121.7 (3)		
			
C8—N1—C4—C5	−0.7 (5)	C11—C12—C13—N2	1.8 (5)
Mn1—N1—C4—C5	−179.2 (3)	C11—C12—C13—C14	−174.8 (3)
N1—C4—C5—C6	0.7 (6)	C18—N3—C14—C15	0.3 (5)
C4—C5—C6—C7	0.0 (5)	Mn1—N3—C14—C15	−179.4 (3)
C5—C6—C7—C8	−0.5 (5)	C18—N3—C14—C13	−179.0 (3)
C4—N1—C8—C7	0.1 (5)	Mn1—N3—C14—C13	1.3 (4)
Mn1—N1—C8—C7	178.8 (3)	N2—C13—C14—N3	−3.4 (4)
C4—N1—C8—C9	−178.9 (3)	C12—C13—C14—N3	173.5 (3)
Mn1—N1—C8—C9	−0.2 (4)	N2—C13—C14—C15	177.4 (3)
C6—C7—C8—N1	0.5 (5)	C12—C13—C14—C15	−5.7 (6)
C6—C7—C8—C9	179.4 (3)	N3—C14—C15—C16	0.5 (5)
C13—N2—C9—C10	0.7 (5)	C13—C14—C15—C16	179.7 (3)
Mn1—N2—C9—C10	171.8 (2)	C14—C15—C16—C17	−1.0 (5)
C13—N2—C9—C8	−177.5 (3)	C15—C16—C17—C18	0.7 (5)
Mn1—N2—C9—C8	−6.4 (4)	C14—N3—C18—C17	−0.6 (5)
N1—C8—C9—N2	4.1 (4)	Mn1—N3—C18—C17	179.1 (3)
C7—C8—C9—N2	−174.9 (3)	C16—C17—C18—N3	0.1 (6)
N1—C8—C9—C10	−174.0 (3)	C12—C11—C19—C20	162.1 (3)
C7—C8—C9—C10	7.0 (5)	C10—C11—C19—C20	−19.3 (5)
N2—C9—C10—C11	0.6 (5)	C12—C11—C19—C24	−18.0 (5)
C8—C9—C10—C11	178.5 (3)	C10—C11—C19—C24	160.6 (3)
C9—C10—C11—C12	−0.7 (5)	C24—C19—C20—C21	−0.7 (6)
C9—C10—C11—C19	−179.4 (3)	C11—C19—C20—C21	179.2 (3)
C10—C11—C12—C13	−0.5 (5)	C19—C20—C21—C22	0.7 (6)
C19—C11—C12—C13	178.2 (3)	C20—C21—C22—C23	−0.2 (6)
C9—N2—C13—C12	−1.9 (5)	C21—C22—C23—C24	−0.3 (6)
Mn1—N2—C13—C12	−173.0 (2)	C22—C23—C24—C19	0.3 (6)
C9—N2—C13—C14	175.2 (3)	C20—C19—C24—C23	0.2 (5)
Mn1—N2—C13—C14	4.1 (4)	C11—C19—C24—C23	−179.7 (3)

### cis-Bromidodicarbonyl(4'-phenyl-2,2':6',2''-terpyridine-κ3N,N',N'')manganese(I) (II) Hydrogen-bond geometry (Å, º)

**Table:**

*D*—H···*A*	*D*—H	H···*A*	*D*···*A*	*D*—H···*A*
C5—H2···Br1^i^	0.95	2.84	3.528 (4)	130
C7—H4···Br1^ii^	0.95	2.86	3.771 (4)	162
C12—H6···Br2^iii^	0.95	2.75	3.688 (7)	171
C12—H6···O2^iii^	0.95	2.55	3.491 (7)	173
C15—H7···Br2^iii^	0.95	2.81	3.759 (7)	175
C15—H7···O2^iii^	0.95	2.50	3.447 (7)	172
C16—H8···Br2^iv^	0.95	2.52	3.286 (7)	138
C16—H8···O2^iv^	0.95	2.57	3.363 (7)	141
C20—H11···Br1^ii^	0.95	2.81	3.743 (4)	168
C20—H11···O3^ii^	0.95	2.55	3.446 (18)	158
C24—H15···Br2^iii^	0.95	2.84	3.611 (7)	139

Symmetry codes: (i) −*x*+1, −*y*, −*z*+2; (ii) *x*, −*y*+1/2, *z*+1/2; (iii) −*x*, *y*+1/2, −*z*+3/2; (iv) *x*, −*y*+1/2, *z*−1/2.
